# Structural insights into the inhibition properties of archaeon citrate synthase from *Metallosphaera sedula*

**DOI:** 10.1371/journal.pone.0212807

**Published:** 2019-02-22

**Authors:** Seul Hoo Lee, Hyeoncheol Francis Son, Kyung-Jin Kim

**Affiliations:** School of Life Sciences (KNU Creative BioResearch Group), KNU Institute for Microorganisms, Kyungpook National University, Daegu, Republic of Korea; Universidade Nova de Lisboa Instituto de Tecnologia Quimica e Biologica, PORTUGAL

## Abstract

*Metallosphaera sedula* is a thermoacidophilic archaeon and has an incomplete TCA/glyoxylate cycle that is used for production of biosynthetic precursors of essential metabolites. Citrate synthase from *M*. *sedula* (*Ms*CS) is an enzyme involved in the first step of the incomplete TCA/glyoxylate cycle by converting oxaloacetate and acetyl-CoA into citrate and coenzyme A. To elucidate the inhibition properties of *Ms*CS, we determined its crystal structure at 1.7 Å resolution. Like other Type-I CS, *Ms*CS functions as a dimer and each monomer consists of two distinct domains, a large domain and a small domain. The oxaloacetate binding site locates at the cleft between the two domains, and the active site was more closed upon binding of the oxaloacetate substrate than binding of the citrate product. Interestingly, the inhibition kinetic analysis showed that, unlike other Type-I CSs, *Ms*CS is non-competitively inhibited by NADH. Finally, amino acids and structural comparison of *Ms*CS with other Type-II CSs, which were reported to be non-competitively inhibited by NADH, revealed that *Ms*CS has quite unique NADH binding mode for non-competitive inhibition.

## Introduction

*Metallosphaera sedula* belongs to the sulfolobaceae family. It is a thermoacidophilic archaea with optimum growth conditions of 73°C and pH 2.0 [1–3]. *M*. *sedula* grows chemolithoautotrophically on metal sulfides or molecular hydrogen, and obtains reducing power by biologically catalyzing iron oxidation and metal sulfide oxidation [2]. *M*. *sedula* gains access to a carbon source by immobilization of bicarbonate using the 3-hydroxypropionate/4-hydroxybutyrate (3-HP/4-HB) cycle [3–5]. The tricarboxylic acid (TCA)/glyoxylate cycle in this strain is incomplete due to the lack of 2-ketoglutarate dehydrogenase, which converts 2-ketoglutarate to succinyl-CoA, and is used for production of biosynthetic precursors including several amino acids and other essential metabolites in this microorganism [6]. Citrate synthase of *M*. *sedula* (*Ms*CS) catalyzes the first step of the incomplete TCA/glyoxylate cycle in this strain.

**Fig 1:**
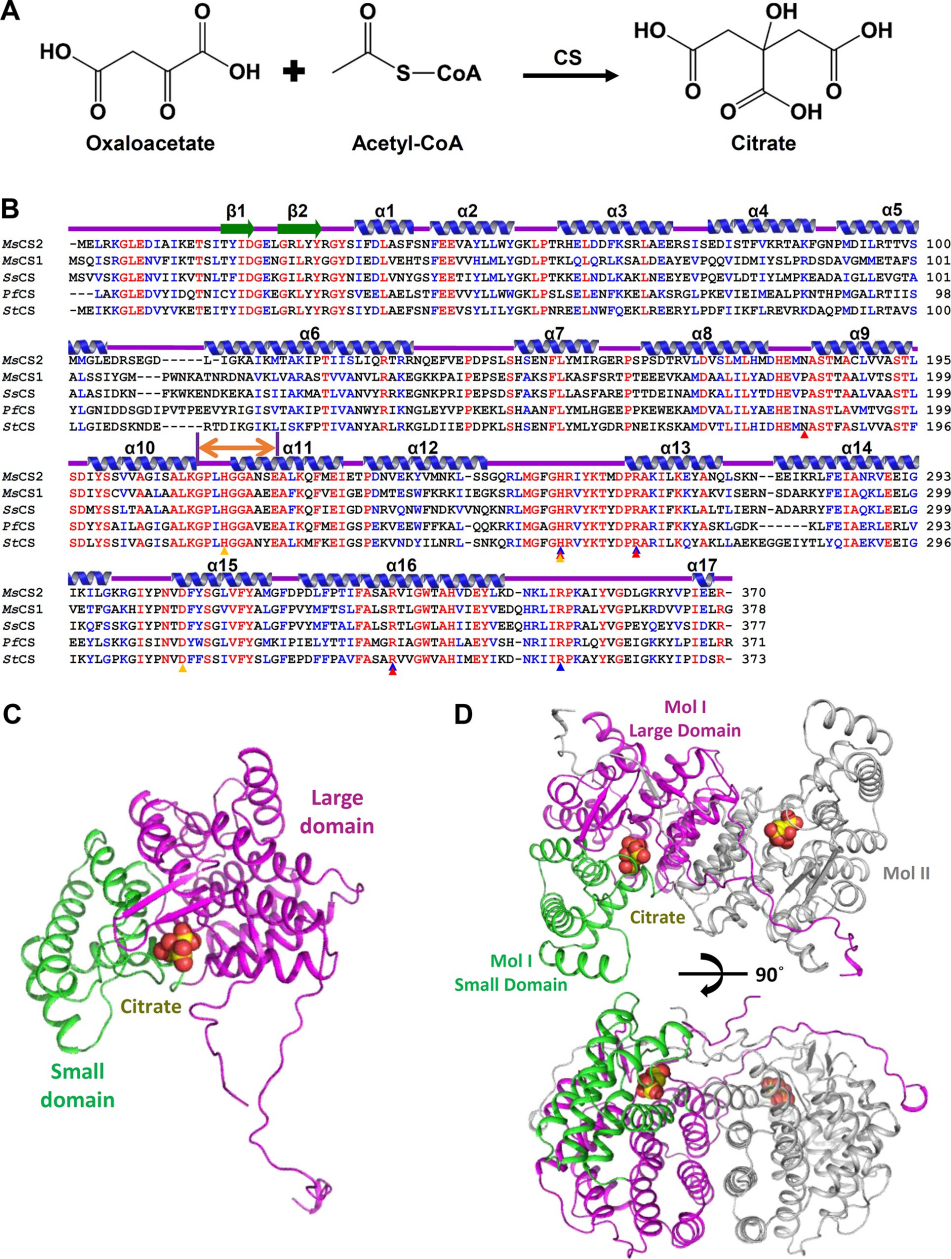
Overall structure of *Ms*CS. **(A)** Scheme of the reaction catalyzed by CS. **(B)** Amino acid sequence alignment of *Ms*CS with other CSs. The secondary structure elements are drawn based on the *Ms*CS structure. The residues involved in the formation of citrate and oxaloacetate binding pocket are indicated by red and blue colored triangles, respectively, and catalytic residues are indicated by yellow. The untangled loop region is shown in the bi-directional orange arrow. *Ms*CS1 and *Ms*CS2 are isoforms of CS in *Metallosphaera sedula*. *Ss*CS, *Pf*CS and *Sd*CS are representatives of CS from *Sulfolobus solfataricus*, *Pyrococcus furiosus* and *Sulfurisphaera tokodaii* respectively. **(C)** The monomeric structure of *Ms*CS. The monomeric structure of *Ms*CS is presented as a cartoon diagram. The large and small domains are distinguished by magenta and green, respectively. The bound citrate product is shown as yellow colored sphere. **(D)** The dimeric structure of *Ms*CS. The dimeric structure of *Ms*CS is shown as a cartoon diagram. The citrate product bound in *Ms*CS is indicated by yellow colored sphere. The large and small domains from a molecule (Mol I) are distinguished by magenta and green colors, respectively, and the other molecular is shown as gray colored cartoon diagram. The bottom figure is rotated 90 degrees horizontally from the above figure.

CSs (EC 2.3.3.1) catalyze conversion of oxaloacetate and acetyl-CoA into citrate and coenzyme A and are well-conserved enzymes in most organisms [7–10] (Fig 1A). In the typical TCA cycle, this reaction is irreversible and is considered a late-limiting step regulated by the ultimate products, which include citrate, ATP and NADH. The reaction proceeds through the non-covalently bound citryl-CoA intermediate in the two-step processes [9]. When the first substrate oxaloacetate binds to its binding site, a domain movement and significant conformational change at the loop region near the substrate binding pocket occur. These alterations induce the formation of an optimal binding pocket for the second substrate, acetyl-CoA [9, 11, 12].

CSs comprise Type-I and Type-II depending on their oligomeric status and an additional β-sheet at the N-terminus. Type-I CSs are homo-dimeric and found in gram-positive bacteria, archaea, and eukaryote [13]. On the other hand, Type-II CSs are mainly found in gram-negative bacteria function as a hexamer and possess an additional β-sheet at their N-terminus [13–15]. Inhibition by NADH is an important biochemical feature that distinguishes these two types of CS. Type-I CSs are regulated by NADH by competitive inhibition, and Type-II CSs are regulated non-competitively with some exceptions [14, 16–19]. Although there have been several structural and biochemical studies on thermal resistance of the archaeal CSs, the enzyme inhibition properties are unclear [20–22]. Thus, it was of interest to understand the inhibition properties of Type-I CSs from archaea that have the incomplete TCA/glyoxylate cycle. In this study, we determined the crystal structures of *Ms*CS in complex with citrate at 1.7 Å and in complex with oxaloacetate at 2.1 Å. The findings reveal that the protein functions as a dimer, similar to other Type-I CSs. Based on the complex structures with its substrate and product, we elucidated the substrate binding site formation and structural changes upon substrate binding. Interestingly, enzyme inhibition kinetic studies revealed that, unlike other Type-I CSs, *Ms*CS is non-competitively inhibited by NADH, which is a unique biochemical property of the archaeal *Ms*CS.

## Materials and methods

### Enzyme preparation of *Ms*CS

The gene coding for *Ms*CS was amplified from chromosomal DNA of *M*. *sedula* by polymerase chain reaction (PCR). The PCR products were digested by NdeI and XhoI restriction enzymes, and sub-cloned into the pET-30a expression vector, which contained a 6×His tag at the C-terminus of the target protein. The resulting expression vector pET-30a:*Ms*CS, was transformed into a *Escherichia coli* BL21(DE3)-T1^R^ strain, which was grown to an OD_600_ of 0.7 in fresh LB medium containing 50 mg L^-1^ kanamycin at 310 K, and *Ms*CS protein expression was induced by 0.5 mM 1-thio-β-D-galatopyranoside (IPTG). After 20 h at 293 K, the cells were harvested by centrifugation at 4,000 × g for 15 min at 277 K. The cell pellet was resuspended in ice-cold buffer A (40 mM Tris-HCl, pH 8.0) and disrupted by ultrasonication. The cell debris was removed by centrifugation at 13,000 × g for 30 min, and the lysate was applied onto a Ni-NTA agarose column (Qiagen). After washing with buffer B (40 mM Tris-HCl, pH 8.0 and 25 mM Imidazole), the bound proteins were eluted with buffer C (40 mM Tris-HCl pH, 8.0 and 300 mM Imidazole). Finally, the trace contaminants were removed by size-exclusion chromatography using a HiPrep 26/60 Sephacryl S-300 HR column (320mL, GE Healthcare Life Sciences) equilibrated with buffer A. The eluted protein had a molecular weight of approximately 85 kDa, indicating a dimeric structure. The protein was concentrated to 54 mg mL^-1^ using spin column (Amicon Ultra Centrifugal Filter, 30 kDa pore size), and kept at 193 K for further experiments. All purification steps were performed at 277 K.

### Crystallization and data collection of *Ms*CS

Crystallization of the purified *Ms*CS protein was initially performed with commercially available sparse-matrix screens, including Index, PEG ion I and II (Hampton Research), and Wizard Classic I and II (Rigaku Reagents) using the sitting-drop vapor diffusion method with MRC Crystallization plate (Molecular Dimensions) at 295 K. Each experiment consisted of mixing 1.0 μL protein solution (54 mg mL^-1^, 40mM Tris-HCl pH 8.0) with 1.0 μL reservoir solution and then equilibrating against 50 μL of reservoir solution. *Ms*CS crystals were observed from several crystallization screening conditions. For crystal improvement, each experiment consisted of mixing 1.0 μL protein solution with 1.0 μL reservoir solution and then equilibrating it against 500 ml of the reservoir solution by the hanging-drop vapor-diffusion method at 295 K. The crystals of the best quality appeared in 0.8 M sodium phosphate monobasic, 1.2 M potassium phosphate dibasic, and 0.1 M sodium acetate pH 4.2. The actual pH condition of 0.8 M sodium phosphate monobasic, 1.2 M potassium phosphate dibasic, and 0.1 M sodium acetate pH 4.2 is pH 6.65. The crystals were transferred to a cryo-protectant solution containing 0.8 M sodium phosphate monobasic, 1.2 M potassium phosphate dibasic, 0.1 M sodium acetate/acetic acid, pH4.2 and 25% (v/v) glycerol. The crystals were harvested with a loop larger than the crystals, and flash-frozen in a nitrogen gas stream at 100 K. Data were collected to a resolution of 1.7 Å at 7A beamline of the Pohang Accelerator Laboratory (PAL, Pohang, Korea), using a Quantum 270 CCD detector (ADSC, USA). All data were indexed, integrated, and scaled together using the HKL2000 software package [23]. Crystals of *Ms*CS in complex with citrate belonged to space group *P*1 with unit cell parameters a = 50.18 Å, b = 53.52 Å, c = 76.28 Å, α = 93.56°, β = 105.73°, and γ = 102.16°. Assuming two *Ms*CS molecules in the asymmetric unit, the crystal volume per unit of protein mass was 2.12 Å^3^ Da^-1^, which indicates a solvent content of approximately 42.15%. *Ms*CS in complex with oxaloacetate was crystallized with the same method except for the addition of 20mM oxaloacetate to the protein solution (50 mg mL^-1^, 40mM Tris-HCl pH 8.0, 20mM oxaloacetate). The crystals of the best quality appeared in 22% polyethylene glycol 3350 and 0.2 M potassium sodium tartrate tetrahydrate supplemented with 20mM of oxaloacetate. The actual pH condition of 22% polyethylene glycol 3350 and 0.2 M potassium sodium tartrate tetrahydrate is pH 7.24. Data were collected to a resolution of 2.0 Å at 7A beamline of the Pohang Accelerator Laboratory (PAL, Pohang, Korea), using a Quantum 270 CCD detector (ADSC, USA). All data were indexed, integrated, and scaled together using the HKL2000 software package. Crystals in complex with oxaloacetate belonged to space group *P*1 with unit cell parameters a = 50.13 Å, b = 55.97 Å, c = 76.97 Å, α = 83.73°, β = 73.94°, and γ = 72.24°. Assuming two molecules of *Ms*CS per asymmetric unit, the crystal volume per unit of protein mass was 2.35 Å^3^ Da^-1^, which corresponds to a solvent content of approximately 48% [24].

### Structure determination of *Ms*CS

The structure of the *Ms*CS in complex with citrate and with oxaloacetate was determined by molecular replacement with the CCP4 [25] version of MOLREP [26], using the structure of CS from *Sulfolbus tokodaii* (PDB code 1VGP) as a search model. Further model building was performed manually using the WinCoot [27], and refinement was performed with CCP4 REFMAC5[27]. The water molecules of model were built by WinCoot. The sigma level was a 0.4 e Å^−3^, 1 sigma at distance between 2.5–3.5 Å under 2Fo-Fc map. The refined models of *Ms*CS in complex with citrate and oxaloacetate were deposited in the Protein Data Bank [28] with PDB codes of 6ABX and 6ABY, respectively.

### Activity assay of *Ms*CS

The activity of *Ms*CS was determined by measuring the increase of absorbance at 412 nm (extinction coefficient of 1.415 × 10^4^ M^-1^ cm^-1^). The enzyme reactions were performed with reaction mixtures of 0.5 mL total volume at 298 K. For the enzymatic activity curve versus pH, relative activity of *Ms*CS was measured under pH 6.0 to pH 10.0. Each reaction mixture contains 10 μM *Ms*CS protein, 50 μM acetyl-CoA, 0.1 mM oxaloacetate, 100 mM buffer (sodium citrate-citric acid pH 6.0–6.5, Tris-HCl pH 7.0–9.0, and Glycine-NaOH pH 9.5–10.0), 150 mM NaCl, and 50 μM DTNB. For the kinetic analysis of oxaloacetate, reaction mixtures containing 0.5 mM acetyl-CoA, 100 mM potassium phosphate, pH 8.0, 150 mM NaCl, 50 μM DTNB, and various concentration of oxaloacetate (0.01 to 5 mM) were used. For the kinetic analysis of acetyl-CoA, reaction mixture containing 100 mM potassium phosphate pH 8.0, 150 mM NaCl, 50 μM DTNB and various concentrations of acetyl-CoA (10 to 200 μM) were used. All reactions were initiated by the addition of enzyme to a final concentration of 100 nM. In order to measure the enzyme inhibition, kinetic analysis was performed using various concentrations of citrate, ATP, and NADH. The kinetic statistics of the enzyme were calculated using Origin software.

## Results and discussion

### Overall structure of *Ms*CS

To understand the molecular mechanism of citrate synthase from *Metallosphaera sedula* (*Ms*CS), we determined its crystal structure at 1.7 Å resolution. The overall refinement and validation statistics are presented in Table 1. Overall, the refined structure present good stereochemistry. The monomeric structure of *Ms*CS consists of seventeen α-helices (α1-α17) and four β-strands (β1-β2), with distinct large domain and small domains (Fig 1B and 1C). The large domain (Met1-His214 and Gly318-Arg370) comprises twelve α-helices (α1-α10 and α16-α17) and two β-strands (β1-β2). The two long α-helices (α6 and α16) located at the center of the large domain are surrounded by the other ten α-helices. Two β-strands (β1 and β2) form a small antiparallel β-sheet. (Fig 1C). The small domain (Gly215-Met317) consists of five α-helices (α11-α15) and constitutes the acetyl-CoA binding pocket. This domain is known to move upon oxaloacetate binding, which will be described in detail later.

**Table 1:** Data collection and refinement statistics.

	*Ms*CS_Citrate	*Ms*CS_Oxaloacetate
**Data collection**		
Wavelength (Å)	0.97934	0.97934
Space group	*P*1	*P*1
Cell dimensions		
*a*, *b*, *c* (Å)	50.2, 53.5, 76.5	50.1, 56.0, 77.0
α, β, γ (°)	93.557, 105.73, 102.16	83.729, 73.941, 72.218
Resolution (Å)	50.00–1.70 (1.73–1.70)	50.00–2.00 (2.03–2.00)
*R*_sym_ or *R*_merge_	5.7 (26.3)	8.7 (30.6)
*I* / σ (*I*)	26.6 (4.5)	29.1 (5.9)
Completeness (%)	97.1 (94.4)	97.2 (96.9)
Redundancy	3.5 (3.2)	1.9 (2.0)
CC^1/2^	0.97 (0.89)	0.97 (0.67)
**Refinement**		
Resolution (Å)	50.00–1.70 (1.73–1.70)	50.00–2.00 (2.03–2.00)
No. reflections	75282 (5256)	48053 (3288)
*R*_work_ / *R*_free_	14.4 / 18.03 (20.5 / 24.6)	14.7 / 19.2 (19.6 / 23.9)
No. atoms	6556	6386
Protein	5919	5970
Ligand/ion	44	88
Water	593	328
*B*-factors	21.07	31.12
Protein	30.17	31.74
Ligand/ion	34.00	49.91
Water	21.15	39.97
R.m.s. deviations		
Bond lengths (Å)	0.014	0.012
Bond angles (°)	1.69	1.51
Planarity (Å)	0.009	0.008
Chirality (Å^3^)	0.16	0.08
Ramachandran plot		
Outliers (%)	0	0
Favored (%)	97.2	97.4
Rotamer outliers (%)	1 (8/662)	2 (15/662)
C^β^ outliers (%)	0.29	0
Clash score	3	3
PDB code	6ABX	6ABY

The structural examination revealed two *Ms*CS molecules and three glycerols in the asymmetric unit, corresponding to a dimeric form of the protein. Dimerization of *Ms*CS is mainly mediated by the large domain (Fig 1D). Four α-helices (α4, α5, α9, and α10) from one molecule interact with the corresponding α-helices from the other molecule (Fig 1D). The long loop region with a short α-helix (α17) at the C-terminus is also important in dimerization via wrapping the α-helical structure of the neighboring molecule. The PISA software [29] computed a buried surface of 4426.9 Å^2^ and the percentage of involved residues is approximately 28.4%. Since archaeal CSs are known to function as a dimer, the dimeric conformation of *Ms*CS indicates that the protein shares the common oligomeric status found in archaeal CS enzymes.

### Active site of *Ms*CS

**Fig 2:**
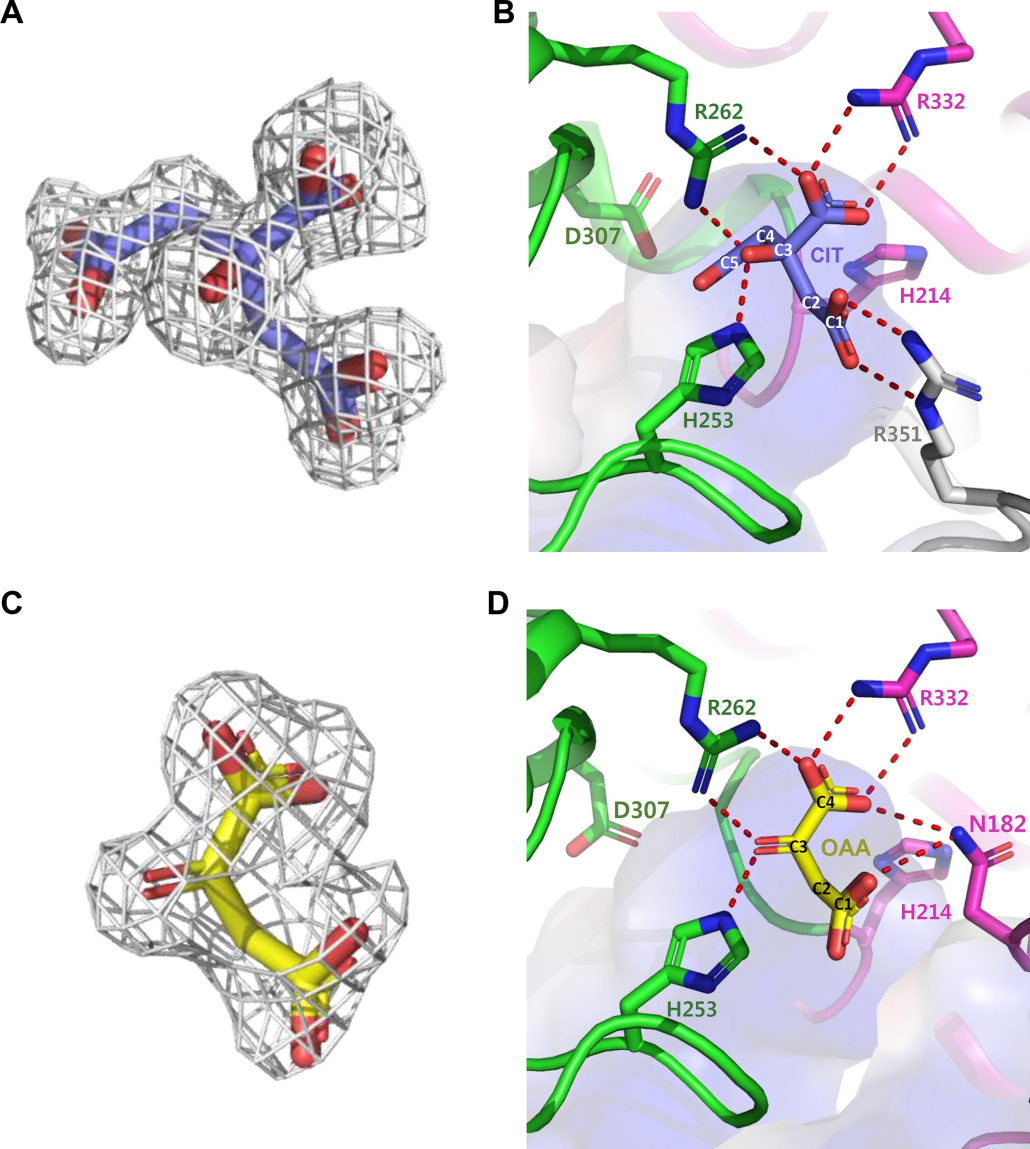
Active site of *Ms*CS. **(A)** Electron density map of the citrate product. The Fo-Fc electron density map is shown with a gray-colored mesh with 2.5 σ contour. The citrate product is shown as a stick model with purple color. **(B)** Citrate binding mode of *Ms*CS. The large and small domains of Mol I are distinguished by magenta and green colors, respectively, and Mol II is shown as and grey color. Residues involved in the formation of citrate binding pocket are shown as a stick model with appropriate labels. The citrate product bound in *Ms*CS is shown as purple stick. The carbon numbers of citrate product are labeled with white color. Red dotted lines indicate polar contacts contributing to the citrate binding. **(C)** Electron density map of the oxaloacetate. The Fo-Fc electron density map of oxaloacetate is shown as a gray-colored mesh with 2.5 σ contour. The oxaloacetate substrate is shown as a stick model with yellow color. **(D)** The binding mode of oxaloacetate substrate. The large and small domains are distinguished magenta and green colors, respectively. Residues involved in the formation of oxaloacetate binding pocket are shown as a stick model with appropriate labels. The oxaloacetate substrate bound in *Ms*CS is shown as a yellow colored stick. The carbon numbers of oxaloacetate substrate are labeled with white. Red dotted lines indicate hydrogen bonds contributing to oxaloacetate substrate binding.

Although we did not add any compound during the purification and crystallization procedure, the citrate product was bound in the structure at pH 6.65 condition (Fig 2A). This provided an explanation of the active site conformation of *Ms*CS. Three conserved catalytic residues, His214, His253, and Asp307, are located near the citrate molecule, indicating that *Ms*CS catalyzes the reaction with a mode identical to other known CSs [10, 30–32] (Fig 2B). The citrate binding pocket is formed at the cleft between the large and small domains. The pocket mainly comprises positively-charged residues, such as arginine and histidine, to accommodate highly negatively-charged molecules. The Arg332 residue from the large domain forms a salt bridge with the C3 carboxyl-group of citrate. His253 residue in the loop region (α10-α11), which connects the large and small domains, forms a hydrogen bonds with the C3 hydroxyl-group, and Arg262 from the small domain is also involved in the stabilization of the C3 carboxyl- and hydroxyl-group of the citrate molecule. Interestingly, the Arg351 residue of the C-terminal long loop from the other molecule is also involved in the stabilization of C2 carboxyl-group of citrate molecule through a salt bridge (Fig 2B).

In order to reveal oxaloacetate binding mode of *Ms*CS, high concentration of oxaloacetate was added to the purified *Ms*CS protein at the crystallization step. *Ms*CS crystals in complex with oxaloacetate were successfully obtained, and we determined its crystal structure at 2.0 Å at pH 7.24 condition (Fig 2C). The structural examination revealed two *Ms*CS molecules and two glycerol molecules in the asymmetric unit. The oxaloacetate binding pocket is almost identical with the citrate binding pocket and is formed by two α-helices (α13 and α16) and three loop regions (α8-α9, α10-α11, and α12-α13). In the large domain, Asn182 interacts with the C2 and C3 carboxyl-groups and Arg332 stabilizes the C3 carboxyl-group of oxaloacetate via a salt bridge. In addition, His253 from the small domain interacts with the C3 carbonyl-group and Arg262 is associated with the stabilization of C3 carbonyl- and carboxyl-groups of oxaloacetate through hydrogen bonds (Fig 2D). Interestingly, Asn182 is not conserved in other CS proteins, and some other CS structures contain Pro at the corresponding position (Fig 1B), indicating that stabilization mode of oxaloacetate might be somewhat different among CSs.

### Conformation change upon product formation

**Fig 3:**
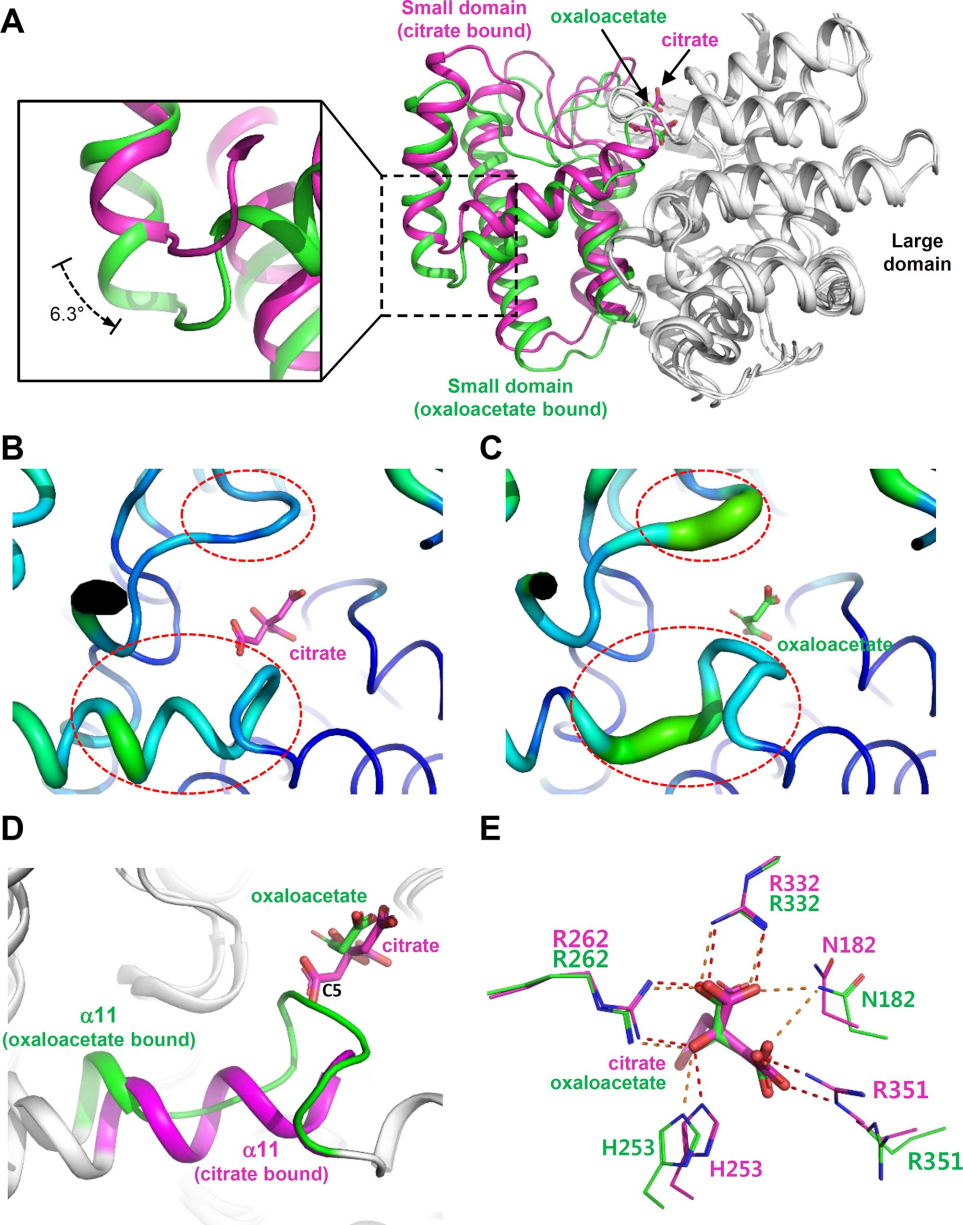
Conformation change upon product formation. **(A)** Structural comparison of citrate and oxaloacetate complex structures. Small domains from citrate and oxaloacetate complex structure are distinguished by magenta and green colors, respectively, and the large domains are shown as a gray color. The left image is a close-up view of the black dotted box to highlight structural movement. **(B, C)** B-factor presentation of *Ms*CS structures of the citrate-binding form **(B)** and oxaloacetate-binding form **(C)**. **(D)** Superposition of α11 helices. Two α11 helices of citrate and oxaloacetate complex structure is distinguished with magenta and green colors, respectively. **(E)** Superposition of the residues involved in the substrate stabilization. Residues from citrate and oxaloacetate complex structures shown as lines with magenta and green colors, respectively. Citrate and oxaloacetate bound to *Ms*CS are indicated by magenta and greed colored sticks, respectively.

CSs undergo domain movement to the closed conformation upon binding of the oxaloacetate substrate, which facilities the formation of an optimal binding pocket for acetyl-CoA [8, 20, 33–35]. It has been also known that the citrate product inhibits the enzyme activity by binding to the substrate binding site, and the binding of citrate to the enzymes induces the closed conformation. However, detailed comparison of the structure of *Ms*CS in complex with oxaloacetate with that in complex with citrate revealed that the conformation of these structures were somewhat different from each other. First the *Ms*CS structure in complex with the citrate product shows a more closed conformation compared with that in complex with the oxaloacetate substrate (Fig 3A). When we superposed these two structures based on the large domain, the small domain of the structure in complex with the citrate product was positioned closer to the large domain (Fig 3A). When we calculate the rotation angle using DynDom [36], the small domain was rotated by 6.3° toward the large domain upon binding of citrate (Fig 3A). Second, the surrounding regions involved in the binding of the citrate product showed much lower B-factor values than those involved in the binding of the oxaloacetate substrate (Fig 3B and 3C), indicating that binding of the citrate product induces a tighter conformation. Third, the region comprising amino acids 212-PLHGGANSE-221 shows an extended α11 in the structure in complex with the citrate product, while the corresponding region in the structure in complex with the oxaloacetate substrate shows an untangled loop (Fig 3D). Since the untangled loop crashes with citrate upon the binding of the citrate product, it seems that the loop moves away from the citrate product forming an extended α-helix (Fig 3D). Finally, we observed differences in residues involved in the binding of these two compounds. While Arg351 interacts with the citrate product, this residue does not interact with the oxaloacetate substrate, but rather rotates away from the compound (Fig 3E). On the contrary, the Asn182 residue does not participate in the binding of citrate but is involved in the binding of oxaloacetate (Fig 3E). Based on these observations, we suggest that binding of the citrate product to the enzyme induces a more compact conformation than that of the oxaloacetate substrate, although both compounds induce domain movement and conformational changes towards the closed conformation.

Here, we need to consider that pHs of the crystallization mixtures for oxaloacetate- and citrate-bound forms were 7.24 and 6.65, respectively, suggesting that the conformational changes described above was possibly affected by changes of pH. Thus, we measured the *Ms*CS activity under various pH conditions to compare the activity between these two pHs. Interestingly, the optimal pH was 9.0, and the activities between pH 7.24 and pH 6.65 were quite different each other (S1 Fig). Based on these observations, we suspect that the conformational differences between the oxaloacetate- and the citrate-bound forms might be also affected by the difference in pH, and further investigations on conformational changes upon the chemicals bound to the enzyme and/or differences in pH are strongly required.

### Inhibition properties analysis of *Ms*CS

**Fig 4:**
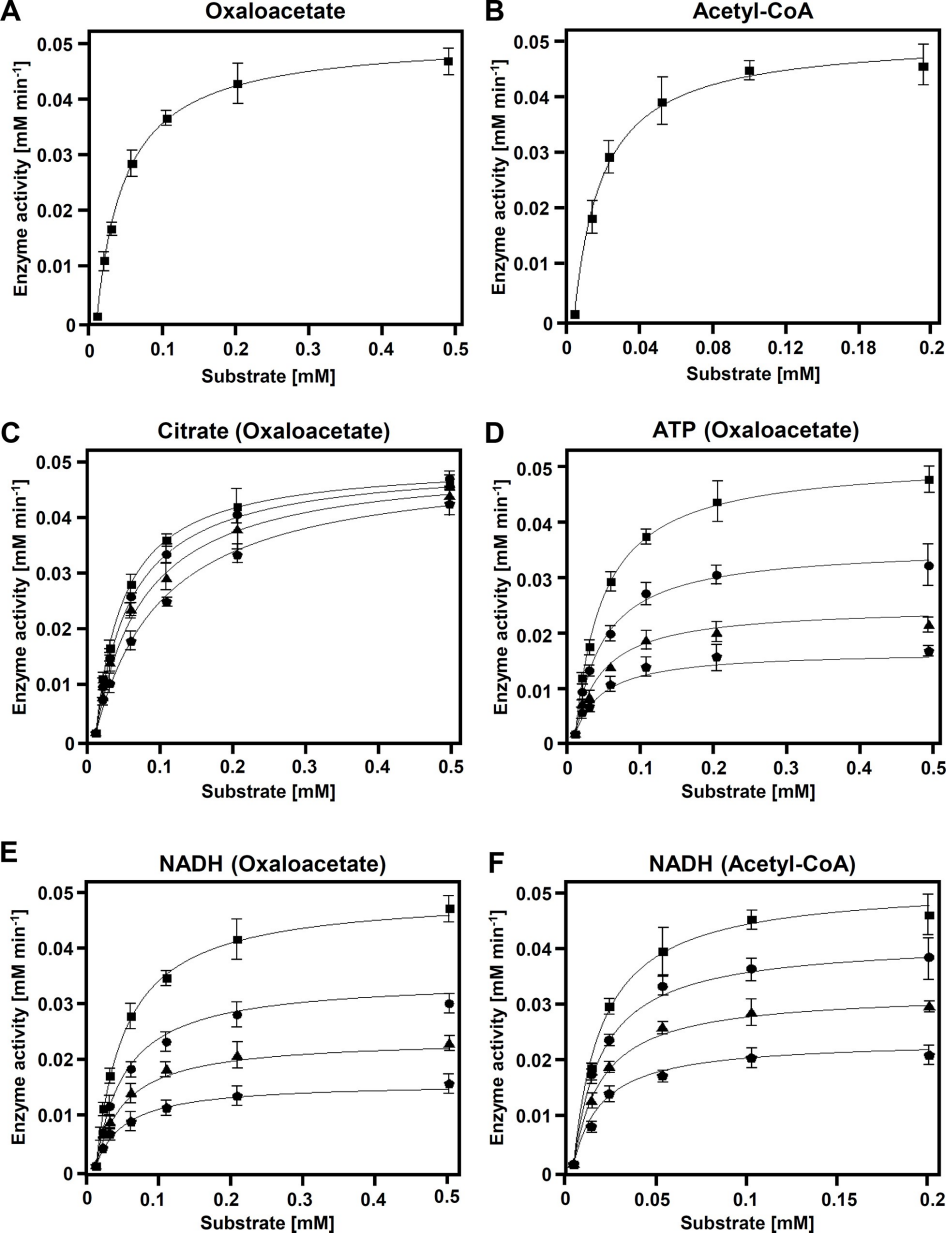
Inhibition properties analysis of *Ms*CS. **(A, B)** Kinetics analysis of *Ms*CS for oxaloacetate **(A)** and acetyl-CoA **(B)**. **(C, D)** Inhibition kinetic analysis to citrate **(C)** and ATP **(D)**. **(E, F)** Inhibition kinetic analysis to NADH. The inhibition kinetics were measured with both oxaloacetate **(E)** and acetyl-CoA **(F)**.

We then performed enzyme kinetic analysis of *Ms*CS using the oxaloacetate and acetyl-CoA substrates. The *K*_*m*_ and *k*_*cat*_ values of oxaloacetate were 0.0414 mM and 7.62 s^-1^, respectively, and those of acetyl-CoA were 0.0165 mM and 8.56 s^-1^, respectively (Fig 4A and 4B, Table 2). Based on these kinetics analyses, the *k*_*cat*_/*K*_*m*_ values of oxaloacetate and acetyl-CoA were 184 and 519 (mM sec)^-1^, respectively. It has been known that the CS enzymes are inhibited by various molecules including citrate, ATP, and NADH [20, 21, 37]. To elucidate the inhibitory properties of archaeon *Ms*CS, we measured inhibition kinetics using citrate, ATP, and NADH. When we measured the inhibition kinetics of citrate, the *K*_*m*_ values increased while the *V*_*max*_ values remained constant, as the concentration of citrate increased (Fig 3C, Table 2). These results indicate that *Ms*CS is competitively inhibited by the citrate product. As we described above, the citrate product binds tightly to the substrate binding site and its binding induces the compact closed conformation, which is consistent with the inhibition kinetic results. We then performed the inhibition kinetic experiment with ATP, and the results showed that the *V*_*max*_ values decreased while the *K*_*m*_ values remained constant, as the concentration of ATP increased (Fig 3D, Table 2), indicating that *Ms*CS is non-competitively inhibited by ATP. Based on these results, we suggest that the archaeon *Ms*CS has the same inhibitory properties against the citrate product and ATP as other conventional Type-I CSs.

**Table 2:** Inhibition kinetics of *Ms*CS.

Inhibitor (varying substrate)	Concentration of inhibitor.	V_max_	Km[mM]	*k*_cat_/K_m_ [mM^-1^ s^-1^]	Type of Inhibition	K_i_ [mM]
Citrate (oxalocetate)	0	0.0456	0.0414	184	Competitive	7.19
	2	0.0470	0.0543	144		
	5	0.0442	0.0612	120		
	10	0.0454	0.0986	76.8		
ATP (oxalocetate)	0	0.0456	0.0414	184	Non-competitive	4.34
	2	0.03032	0.03702	136		
	5	0.01959	0.03741	87.3		
	10	0.01466	0.04022	60.7		
NADH (oxalocetate)	0	0.0456	0.0414	184	Non-competitive	4.41
	2	0.0283	0.0411	115		
	5	0.0203	0.0384	88.1		
	10	0.0137	0.0422	54.1		
NADH (acetyl-CoA)	0	0.0513	0.0165	519	Non-competitive	7.96
	2	0.0412	0.0152	452		
	5	0.0321	0.0162	331		
	10	0.0229	0.0178	214		

**Fig 5:**
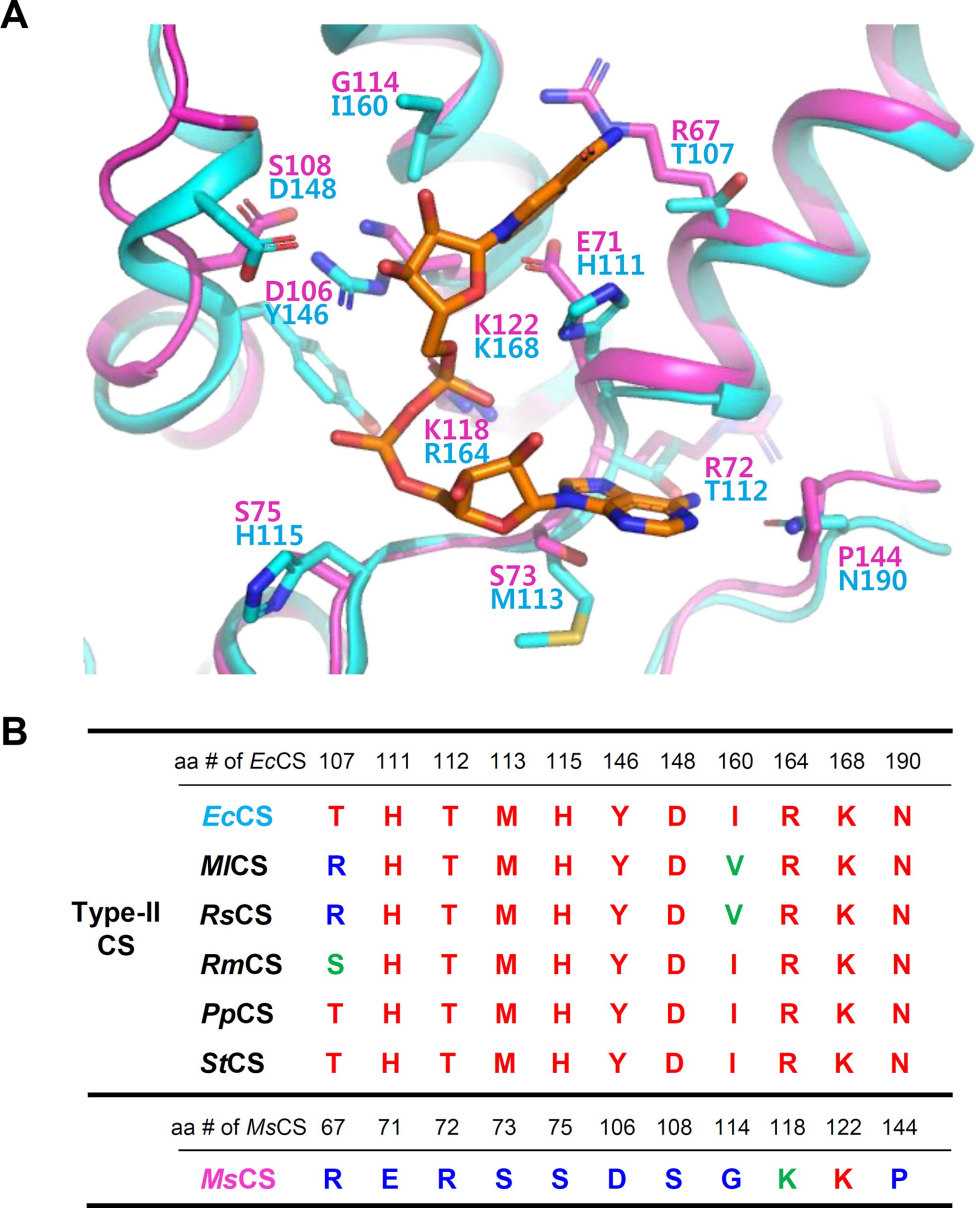
Structural and amino acid comparison of *Ms*CS with other Type-II CSs. **(A)** Superposition of *Ms*CS with CS from *Escherichia coli* (*Ec*CS) in complex with the NADH inhibitor. The *Ms*CS and *Ec*CS are distinguished as magenta and light-blue colors, respectively. **(B)** Amino acid sequence alignment of key residues involved in NADH binding in *Ec*CS and several Type-II CSs. *Ml*CS, *Rs*CS, *Rm*CS, *Pp*CS and *St*CS are representatives of CS from *Methylomicrobium album*, *Rhodobacter sphaeroides*, *Ralstonia metallidurans*, *Pseudomonas putida*, and *Salmonella typhimurium*, respectively. The colored amino acid are indicated to same (red), similar (green) and different (blue).

We also performed inhibition kinetic experiment using NADH. The *V*_*max*_ values were decreased while the *K*_*m*_ values stayed constant, as the concentration of NADH increased (Fig 4B, Table 2). This phenomenon was observed when both oxaloacetate and acetyl-CoA were used as a variable substrate (Fig 4B, Table 2), and these results indicate that *Ms*CS is non-competitively inhibited by NADH. Interestingly, non-competitive inhibition by NADH has been only reported from some Type-II CSs with a conserved NADH binding motif that is allosterically regulated, and most Type-I CSs are known to be competitively inhibited by NADH [13–15, 38–41]. Thus, the non-competitive inhibition of *Ms*CS by NADH seems to be a quite unique inhibitory property of the archaeon enzyme. The crystal structure of the Type-II CS from *Escherichia coli* (*Ec*CS) in complex with the NADH inhibitor elucidated the binding mode of the NADH inhibitor (Fig 5A) [39]. Moreover, the residues involved in the NADH binding of *Ec*CS are highly conserved among other Type-II CSs, such as *Ml*CS, *Rs*CS, *Rm*CS, *Pp*CS, and *St*CS, which are non-competitively inhibited by NADH (Fig 5B) [39]. However, amino acid and structural comparisons of *Ms*CS with *Ec*CS in complex with the NADH showed that the residues located at the NADH binding site of *Ec*CS were almost completely different from those located at the corresponding positions in *Ms*CS (Fig 5A and 5B). These observations indicate that NADH might bind to *Ms*CS at a different site from other Type-II CSs, although *Ms*CS is non-competitively inhibited by NADH as other Type-II CSs. Our attempts to determine the *Ms*CS structure in complex with NADH have failed and further structural studies will be necessary to reveal the binding mode of NADH in *Ms*CS.

In summary, in order to elucidate the molecular mechanism of *Ms*CS, we determined its crystal structure in complex with oxaloacetate and citrate. The structural information revealed that *Ms*CS is inhibited by citrate through conformational change. We also performed kinetic analyses to verify the inhibition properties of *Ms*CS, which showed that *Ms*CS is inhibited by citrate and ATP, like other known CSs. Interestingly, *Ms*CS is also inhibited non-competitively by NADH even though it belongs to Type-I CS with a dimeric structure. Furthermore, by comparing *Ms*CS with Type-II CSs reported to be inhibited by NADH, *Ms*CS was predicted to have an inhibition mode of NADH that differs from Type-II CSs.

## Supporting information

S1 Fig (PDF)

## References

[1] Auernik KS, Cooper CR, Kelly RM. Life in hot acid: pathway analyses in extremely thermoacidophilic archaea. Curr Opin Biotechnol. 2008;19(5):445–53. Epub 2008/09/02. 10.1016/j.copbio.2008.08.001 18760359 PMC3771398

[2] Auernik KS, Kelly RM. Physiological versatility of the extremely thermoacidophilic archaeon Metallosphaera sedula supported by transcriptomic analysis of heterotrophic, autotrophic, and mixotrophic growth. Appl Environ Microbiol. 2010;76(3):931–5. Epub 2009/12/17. 10.1128/AEM.01336-09 20008169 PMC2813022

[3] Chuakrut S, Arai H, Ishii M, Igarashi Y. Characterization of a bifunctional archaeal acyl coenzyme A carboxylase. J Bacteriol. 2003;185(3):938–47. Epub 2003/01/21. 10.1128/JB.185.3.938-947.2003 12533469 PMC142822

[4] Estelmann S, Hugler M, Eisenreich W, Werner K, Berg IA, Ramos-Vera WH, et al Labeling and enzyme studies of the central carbon metabolism in Metallosphaera sedula. J Bacteriol. 2011;193(5):1191–200. Epub 2010/12/21. 10.1128/JB.01155-10 21169486 PMC3067578

[5] Berg IA, Kockelkorn D, Ramos-Vera WH, Say RF, Zarzycki J, Hugler M, et al Autotrophic carbon fixation in archaea. Nat Rev Microbiol. 2010;8(6):447–60. Epub 2010/05/11. 10.1038/nrmicro2365 . 20453874

[6] Hawkins AB, Adams MW, Kelly RM. Conversion of 4-hydroxybutyrate to acetyl coenzyme A and its anapleurosis in the Metallosphaera sedula 3-hydroxypropionate/4-hydroxybutyrate carbon fixation pathway. Appl Environ Microbiol. 2014;80(8):2536–45. Epub 2014/02/18. 10.1128/AEM.04146-13 24532060 PMC3993168

[7] Krebs H. Citric acid cycle: a chemical reaction for life. Nurs Mirror. 1979;149(7):30–2. Epub 1979/08/16. . 257627

[8] Remington SJ. Structure and mechanism of citrate synthase. Curr Top Cell Regul. 1992;33:209–29. Epub 1992/01/01. . 1499334 10.1016/b978-0-12-152833-1.50017-4

[9] Wiegand G, Remington SJ. Citrate synthase: structure, control, and mechanism. Annu Rev Biophys Biophys Chem. 1986;15:97–117. Epub 1986/01/01. 10.1146/annurev.bb.15.060186.000525 . 3013232

[10] Karpusas M, Branchaud B, Remington SJ. Proposed mechanism for the condensation reaction of citrate synthase: 1.9-A structure of the ternary complex with oxaloacetate and carboxymethyl coenzyme A. Biochemistry. 1990;29(9):2213–9. Epub 1990/03/06. . 2337600

[11] Wiegand G, Remington S, Deisenhofer J, Huber R. Crystal structure analysis and molecular model of a complex of citrate synthase with oxaloacetate and S-acetonyl-coenzyme A. J Mol Biol. 1984;174(1):205–19. Epub 1984/03/25. . 6716477 10.1016/0022-2836(84)90373-5

[12] Remington S, Wiegand G, Huber R. Crystallographic refinement and atomic models of two different forms of citrate synthase at 2.7 and 1.7 A resolution. J Mol Biol. 1982;158(1):111–52. Epub 1982/06/15. . 7120407 10.1016/0022-2836(82)90452-1

[13] Nguyen NT, Maurus R, Stokell DJ, Ayed A, Duckworth HW, Brayer GD. Comparative analysis of folding and substrate binding sites between regulated hexameric type II citrate synthases and unregulated dimeric type I enzymes. Biochemistry. 2001;40(44):13177–87. 10.1021/bi010408o WOS:000172057100007. 11683626

[14] Danson MJ, Weitzman PD. Thiol groups of Escherichia coli citrate synthase and their influence on activity and regulation. Biochim Biophys Acta. 1977;485(2):452–64. Epub 1977/12/08. . 200273 10.1016/0005-2744(77)90181-4

[15] Francois JA, Starks CM, Sivanuntakorn S, Jiang H, Ransome AE, Nam JW, et al Structure of a NADH-insensitive hexameric citrate synthase that resists acid inactivation. Biochemistry. 2006;45(45):13487–99. Epub 2006/11/08. 10.1021/bi061083k . 17087502

[16] Bayer E, Bauer B, Eggerer H. Evidence from inhibitor studies for conformational changes of citrate synthase. Eur J Biochem. 1981;120(1):155–60. Epub 1981/11/01. . 7308213 10.1111/j.1432-1033.1981.tb05683.x

[17] Smith CM, Williamson JR. Inhibition of citrate synthase by succinyl-CoA and other metabolites. FEBS Lett. 1971;18(1):35–8. Epub 1971/10/15. . 11946076 10.1016/0014-5793(71)80400-3

[18] Jangaard NO, Unkeless J, Atkinson DE. The inhibition of citrate synthase by adenosine triphosphate. Biochim Biophys Acta. 1968;151(1):225–35. Epub 1968/01/08. . 4867623 10.1016/0005-2744(68)90177-0

[19] Hostalek Z, Ryabushko TA, Cudlin J, Vanek Z. Regulation of biosynthesis of secondary metabolites. IV. Inhibition of citrate synthase in Streptomyces aureofaciens by adenosine triphosphate. Folia Microbiol (Praha). 1969;14(2):121–7. Epub 1969/01/01. . 5768837 10.1007/BF02892880

[20] Russell RJ, Ferguson JM, Hough DW, Danson MJ, Taylor GL. The crystal structure of citrate synthase from the hyperthermophilic archaeon pyrococcus furiosus at 1.9 A resolution. Biochemistry. 1997;36(33):9983–94. Epub 1997/08/19. 10.1021/bi9705321 . 9254593

[21] Bell GS, Russell RJ, Connaris H, Hough DW, Danson MJ, Taylor GL. Stepwise adaptations of citrate synthase to survival at life's extremes. From psychrophile to hyperthermophile. Eur J Biochem. 2002;269(24):6250–60. Epub 2002/12/11. . 12473121 10.1046/j.1432-1033.2002.03344.x

[22] Boutz DR, Cascio D, Whitelegge J, Perry LJ, Yeates TO. Discovery of a thermophilic protein complex stabilized by topologically interlinked chains. J Mol Biol. 2007;368(5):1332–44. Epub 2007/03/31. 10.1016/j.jmb.2007.02.078 17395198 PMC1955483

[23] Otwinowski Z, Minor W. Processing of X-ray diffraction data collected in oscillation mode. Methods Enzymol. 1997;276:307–26. Epub 1997/01/01. . 27754618 10.1016/S0076-6879(97)76066-X

[24] Chruszcz M, Potrzebowski W, Zimmerman MD, Grabowski M, Zheng H, Lasota P, et al Analysis of solvent content and oligomeric states in protein crystals—does symmetry matter? Protein Sci. 2008;17(4):623–32. Epub 2008/03/25. 10.1110/ps.073360508 18359856 PMC2271157

[25] Collaborative Computational Project N. The CCP4 suite: programs for protein crystallography. Acta Crystallogr D Biol Crystallogr. 1994;50(Pt 5):760–3. Epub 1994/09/01. 10.1107/S0907444994003112 . 15299374

[26] Vagin A, Teplyakov A. Molecular replacement with MOLREP. Acta Crystallogr D Biol Crystallogr. 2010;66(Pt 1):22–5. Epub 2010/01/09. 10.1107/S0907444909042589 . 20057045

[27] Murshudov GN, Vagin AA, Dodson EJ. Refinement of macromolecular structures by the maximum-likelihood method. Acta Crystallogr D Biol Crystallogr. 1997;53(Pt 3):240–55. Epub 1997/05/01. 10.1107/S0907444996012255 . 15299926

[28] Sussman JL. Protein data bank deposits. Science. 1998;282(5396):1993 Epub 1999/01/05. . 9874649 10.1126/science.282.5396.1991f

[29] Krissinel E, Henrick K. Inference of macromolecular assemblies from crystalline state. J Mol Biol. 2007;372(3):774–97. 10.1016/j.jmb.2007.05.022 . 17681537

[30] Aleksandrov A, Zvereva E, Field M. The mechanism of citryl-coenzyme A formation catalyzed by citrate synthase. J Phys Chem B. 2014;118(17):4505–13. Epub 2014/04/12. 10.1021/jp412346g . 24720842

[31] Alter GM, Casazza JP, Zhi W, Nemeth P, Srere PA, Evans CT. Mutation of essential catalytic residues in pig citrate synthase. Biochemistry. 1990;29(33):7557–63. Epub 1990/08/21. . 1702991 10.1021/bi00485a003

[32] Evans CT, Kurz LC, Remington SJ, Srere PA. Active site mutants of pig citrate synthase: effects of mutations on the enzyme catalytic and structural properties. Biochemistry. 1996;35(33):10661–72. Epub 1996/08/20. 10.1021/bi960336e . 8718855

[33] Russell RJ, Gerike U, Danson MJ, Hough DW, Taylor GL. Structural adaptations of the cold-active citrate synthase from an Antarctic bacterium. Structure. 1998;6(3):351–61. Epub 1998/04/29. . 9551556 10.1016/s0969-2126(98)00037-9

[34] Russell RJ, Hough DW, Danson MJ, Taylor GL. The crystal structure of citrate synthase from the thermophilic archaeon, Thermoplasma acidophilum. Structure. 1994;2(12):1157–67. Epub 1994/12/15. . 7704526 10.1016/s0969-2126(94)00118-9

[35] Kanamori E, Kawaguchi S, Kuramitsu S, Kouyama T, Murakami M. Structural comparison between the open and closed forms of citrate synthase from Thermus thermophilus HB8. Biophys Physicobiol. 2015;12:47–56. Epub 2015/01/01. 10.2142/biophysico.12.0_47 27493854 PMC4736845

[36] Hayward S, Berendsen HJ. Systematic analysis of domain motions in proteins from conformational change: new results on citrate synthase and T4 lysozyme. Proteins. 1998;30(2):144–54. Epub 1998/03/07. . 9489922

[37] Murphy JR, Donini S, Kappock TJ. An active site-tail interaction in the structure of hexahistidine-tagged Thermoplasma acidophilum citrate synthase. Acta Crystallogr F Struct Biol Commun. 2015;71(Pt 10):1292–9. Epub 2015/10/13. 10.1107/S2053230X15015939 26457521 PMC4601594

[38] Selwood T, Jaffe EK. Dynamic dissociating homo-oligomers and the control of protein function. Arch Biochem Biophys. 2012;519(2):131–43. Epub 2011/12/21. 10.1016/j.abb.2011.11.020 22182754 PMC3298769

[39] Maurus R, Nguyen NT, Stokell DJ, Ayed A, Hultin PG, Duckworth HW, et al Insights into the evolution of allosteric properties. The NADH binding site of hexameric type II citrate synthases. Biochemistry. 2003;42(19):5555–65. Epub 2003/05/14. 10.1021/bi020622s . 12741811

[40] Harford S, Weitzman PD. Evidence of isosteric and allosteric nucleotide inhibition of citrate synthease from multiple-inhibition studies. Biochem J. 1975;151(2):455–8. Epub 1975/11/01. 175782 10.1042/bj1510455 PMC1172380

[41] Lohlein-Werhahn G, Goepfert P, Eggerer H. Purification and properties of an archaebacterial enzyme: citrate synthase from Sulfolobus solfataricus. Biol Chem Hoppe Seyler. 1988;369(2):109–13. Epub 1988/02/01. . 3130075 10.1515/bchm3.1988.369.1.109

