# Supplementary material for: Structural insights into the inhibition properties of archaeon citrate synthase from Metallosphaera sedula
Source: PLoS One. 2019 Feb 22;14(2):e0212807. doi: 10.1371/journal.pone.0212807 (PMC6386500; doi:10.1371/journal.pone.0212807)
Supplement: S1 Fig — (PDF) [file pone.0212807.s001.pdf]

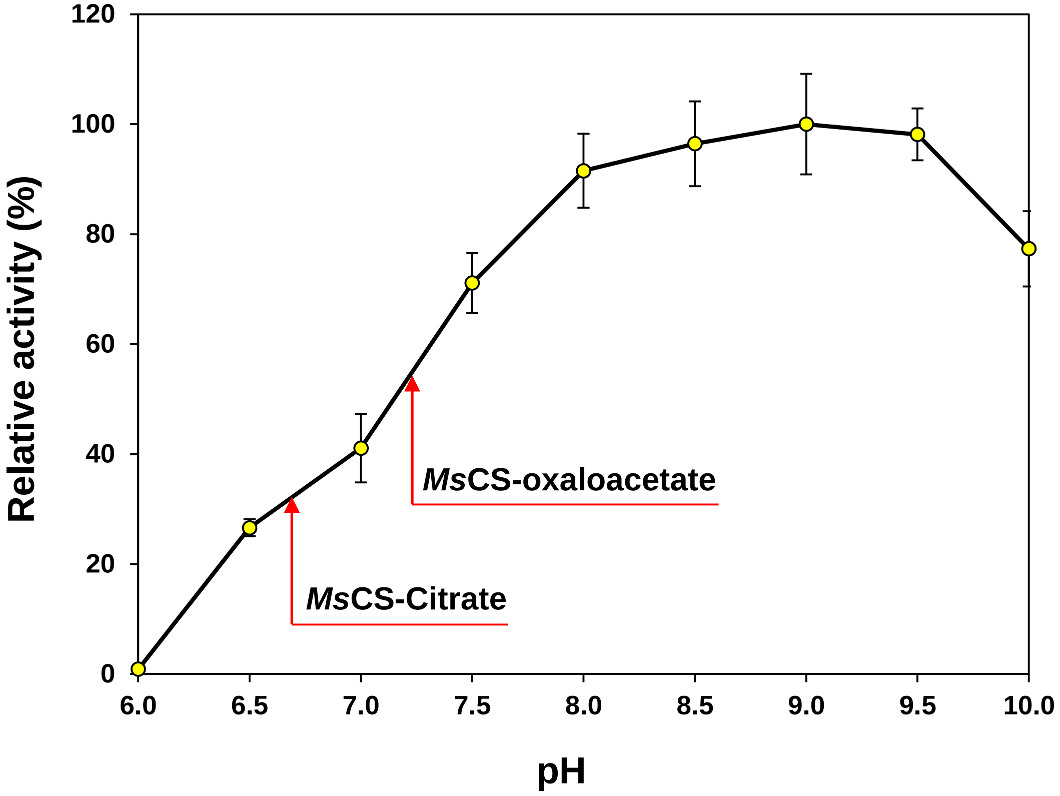

S1 Fig. Enzymatic activity curve versus pH. Relative activity (%) of *Ms*CS was measured at pH 6.0 to 10.0. Each pH of the crystallization mixtures for oxaloacetate-bound forms (7.24) and citrate-bound forms (6.65) are indicated by the arrow which color is red, respectively.
